# Role of Lipid-Lowering Therapy in Peripheral Artery Disease

**DOI:** 10.3390/jcm11164872

**Published:** 2022-08-19

**Authors:** Agastya D. Belur, Aangi J. Shah, Salim S. Virani, Mounica Vorla, Dinesh K. Kalra

**Affiliations:** 1 Division of Cardiology, University of Louisville, Louisville, KY 40202, USA; 2 Department of Internal Medicine, University of Louisville, Louisville, KY 40292, USA; 3 Section of Cardiovascular Research, Baylor College of Medicine and Section of Cardiology, Michael E. DeBakey Veterans Affairs Medical Center, Houston, TX 77030, USA

**Keywords:** atherosclerosis, peripheral artery disease, intermittent claudication, amputation, critical limb ischemia, lipoprotein, statin, PCSK9 inhibitors, icosapent ethyl, inclisiran

## Abstract

Atherosclerosis is a multifactorial, lipoprotein-driven condition that leads to plaque formation within the arterial tree, leading to subsequent arterial stenosis and thrombosis that accounts for a large burden of cardiovascular morbidity and mortality globally. Atherosclerosis of the lower extremities is called peripheral artery disease and is a major cause of loss in mobility, amputation, and critical limb ischemia. Peripheral artery disease is a common condition with a gamut of clinical manifestations that affects an estimated 10 million people in the United States of America and 200 million people worldwide. The role of apolipoprotein B-containing lipoproteins, such as LDL and remnant lipoproteins in the development and progression of atherosclerosis, is well-established. The focus of this paper is to review existing data on lipid-lowering therapies in lower extremity atherosclerotic peripheral artery disease.

## 1. Introduction

Acute and chronic arterial atherothrombosis accounts for a high burden of cardiovascular (CV) morbidity and mortality globally [1,2]. Atherosclerosis affects arteries of all sizes and is the consequence of oxidized lipids that become entrapped in the extracellular matrix of the subendothelial space [3]. Arterial branch points and sites along the inner curvature of arteries that have low or oscillatory endothelial shear stress often serve as a nidus for atheroma formation. Atherosclerosis of the lower extremities is called peripheral artery disease (PAD). Although other arterial diseases can also cause ischemia of the legs (such as thromboangiitis obliterans, embolism, fibromuscular dysplasia, and vasculitides), atherosclerotic occlusion is the commonest cause of PAD in the US.

PAD has a gamut of clinical manifestations, ranging from asymptomatic disease to acute or chronic symptoms due to a combination of ischemia, thrombosis, and/or embolism. Chronic PAD can have a few different subtypes, such as (i) symptomatic disease with classic symptoms of intermittent claudication (IC), often diagnosed by an abnormal ankle brachial index (ABI) value of <0.9; (ii) asymptomatic PAD with either a normal or abnormal ABI; or (iii) having had a prior lower extremity (LE) arterial revascularization procedure. The leading cause of morbidity in PAD is loss in mobility and ambulation due to IC, which is ischemia-induced discomfort, cramping, heaviness, or frank pain in the affected limb precipitated by physical activity and relieved by rest. Patients are usually able to reliably relate what distance they can walk before IC appears, and this is called the claudication distance. As the disease advances, the claudication distance diminishes. In severe disease, chronic limb-threatening ischemia (CLTI) is the most feared complication and is associated with limb loss and mortality [4]. Other complications of PAD that add to morbidity and mortality include the development of arterial ulcers, gangrene, poor wound healing, and deep soft tissue and bone infections. Regardless of symptoms, PAD is a strong harbinger of associated current or future CV disease and major adverse CV events (MACE). As an example, stroke and myocardial infarction (MI) are three and four times more likely, respectively, in patients suffering from PAD [5,6,7]. A reduced ABI has a two-fold higher risk of MI, angina, congestive heart failure, and cerebrovascular disease [8]; overall mortality in PAD correlates strongly with a decline in the ABI [9].

## 2. Role of Lipids in Atherosclerosis

**Figure 1:**
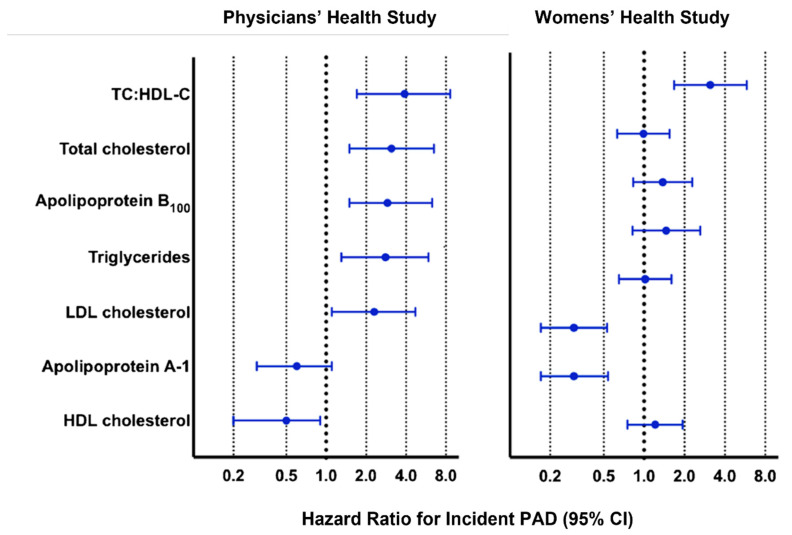
**Left panel**: Adjusted hazard ratio of lipids and apolipoproteins and PAD in the Physicians’ Health Study. Note relative risk and 95% confidence intervals for the top and bottom quartile of various lipid and lipoprotein levels after adjustment for smoking status, age, body mass index, frequency of exercise, presence of diabetes and hypertension, and family history of premature atherosclerotic disease. **Right panel**: Women’s Health Study with hazard ratios and 95% confidence intervals for the top versus bottom tertile of various lipid and lipoprotein levels after adjustment for number of packs smoked over the years, age, body mass index, high-sensitivity C-reactive protein, and presence of hypertension, metabolic syndrome, prior lipid-lowering therapy, and hormonal therapy [12].

**Figure 2:**
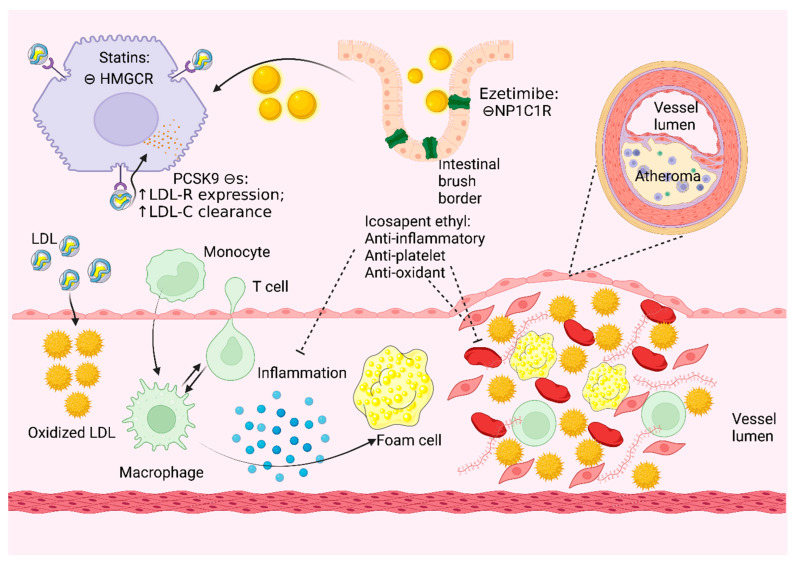
Role of oxidized lipoproteins in the development of atherosclerosis, with specific targets and mechanism of action of statins, ezetimibe, and PCSK9 inhibitors. HMGCR: 3-β-Hydroxy β-methylglutaryl-CoA reductase, LDL: Low-density lipoprotein, LDL-C: Low-density lipoprotein cholesterol, LDL-R: Low-density lipoprotein receptor, NP1C1R: Niemann–Pick C1-like 1 receptor, PCSK9: Proprotein Convertase Subtilisin/Kexin Type 9.

Atherosclerosis is a multifactorial, lipoprotein-mediated condition that leads to plaque formation at vulnerable sites of arteries through inflammation, necrosis, fibrosis, and calcification over decades. Endothelial injury with subsequent accumulation of apolipoprotein B-containing lipoproteins, such as LDL and remnant lipoproteins, is the first step in the formation of atherosclerotic plaque in arteries (Figure 1) [10]. Hyperlipidemia can directly impair endothelial cell function by increasing free radical production, which accelerates the oxidation of cholesterol deposits in the endothelium. Oxidized lipoproteins are ingested by macrophages through scavenger receptors and accumulate in phagocytes that then transform into foam cells. Oxidized lipoproteins also stimulate the release of growth factors, cytokines, and chemokines that increase the recruitment of inflammatory cells into atherosclerotic lesions. Oxidized LDL exerts cytotoxic effects on endothelial cells and smooth muscle cells, which further potentiates endothelial cell dysfunction (Figure 2). Recent studies have explored the role of inflammation in the initiation and progression of plaque, such as neutrophils, normal-density granulocytes, and low-density granulocytes, especially in patients with chronic inflammatory disease states [11]. Atherosclerotic plaques may progress via fibrosis or calcification over decades, and eventually, a vulnerable plaque may undergo rupture, ulceration, or erosion, which then leads to acute thrombosis in the affected artery with catastrophic outcomes, such as MI, stroke, or CLTI.

## 3. Epidemiology and Clinical Burden of PAD

PAD is estimated to affect 10–25% people aged ≥55 years, and the prevalence increases with age to approximately 40% among people >80 years [13]. An estimated 8 to 10 million people in the USA and 200 million people worldwide suffer from PAD [14,15]. Males have a higher incidence of PAD compared to females, and black individuals are more likely to develop PAD than non-Hispanic white individuals [16]. However, despite efforts to increase screening for asymptomatic PAD with ABI and questionnaires to detect IC, the prevalence of this disease varies according to the population studied with underdiagnosis among Hispanic individuals and other racial and ethnic minorities [14,16]. The overall estimated prevalence of IC ranges from 1–4.5% in a population older than 40 years [6,17]. Approximately 10–30% of patients with PAD have some degree of IC. The prevalence and incidence of CLTI increase with age and are greater in men [16], with an incidence of 22 per 100,000 per year and affecting 1–2% of all patients with PAD. There is less data available on the incidence and prevalence of acute limb ischemia (ALI), with estimates of approximately 1–2% among patients with {Singh, 2021, Prescribing of Statins After Lower Extremity Revascularization Procedures in the US} symptomatic PAD [18]. CLTI has a mortality rate of ~25% in the first year after initial presentation and an estimated three-year limb loss rate as high as 40%, with five-year survival rate of <30%. Diabetic patients have a higher incidence of adverse outcomes and amputations [4,19]. Approximately one quarter of all patients with CLTI are not considered candidates for vascular surgery or endovascular procedures, and amputation is often the only option available. This corresponds to 120,000 amputations annually in the United States [20,21], with the overall incidence of amputation ranging from 112 to 250 per million per year. There is an association between recurrent CLTI due to the failure of LE revascularization and worse patient outcomes [22], and the bypass versus angioplasty in severe ischemia of the leg (BASIL) trial [23] demonstrated a re-intervention rate among 216 patients with CLTI treated by percutaneous transluminal angioplasty as high as 26% at one year. The increasing number of patients with CLTI and its overall poor prognosis has led to a higher need for novel therapies to induce angiogenesis, with the most emphasis being placed on gene and cell therapy.

PAD is often treated using a multifaceted approach, and treatment options depend on lipid levels, limb function, severity of symptoms, and presence of comorbidities, such as diabetes, smoking, and hypertension. Conservative medical management involves an exercise regimen and use of antiplatelet agents, antithrombotic drugs, and phosphodiesterase-3 inhibitors, such as cilostazol, when appropriate. Regardless of symptoms, all patients must receive lipid lowering therapy to reach goal lipid levels discussed below and should be counseled on a heart-healthy, low-fat diet. Complications of PAD and CLTI are often treated with invasive and expensive therapies that are associated with high morbidity and mortality directly and indirectly through limb loss, need for revascularization, and failure of conservative treatment leading to amputation. Early diagnosis and treatment of PAD is imperative in preventing complications that are frequently life- and limb-threatening.

## 4. Role of Statins in PAD

### 4.1. Mechanism of Action of Statins

Statins are recommended as first-line therapy in the treatment of patients with PAD to decrease the lipid plaque burden and reduce the risk of adverse CV events [6,7]. Statins competitively inhibit 3-β-Hydroxy β-methylglutaryl-CoA reductase, which is an enzyme responsible for the synthesis of mevalonic acid. This is a rate-limiting step in cholesterol synthesis. Inhibition of cholesterol synthesis causes an upregulation of LDL receptors on the surface of hepatocytes, thereby promoting greater hepatic uptake of LDL and reducing the circulating atherogenic low-density lipoprotein cholesterol (LDL-C) burden. In addition to promoting plaque stability, statins have also been associated with anti-inflammatory effects by inhibiting neointimal hyperplasia and vascular smooth muscle cell proliferation and increasing the release of nitric oxide, which allows for vasodilation and decreased platelet aggregation [24,25]. These pleiotropic effects of statins likely play a part in reducing downstream events by promoting plaque stability and some degree of plaque regression [26].

### 4.2. Clinical Outcomes Associated with Statin Use in Patients with PAD

One of the earliest studies involving statins was the Scandinavian Simvastatin Survival Study (4S) [27], a large clinical trial in which 4444 adults aged 35 to 70 years with prior MI or angina pectoris and with total serum cholesterol between 212.7 and 309.4 mg/dL were randomized to receive either a placebo or simvastatin 20–40 mg/day. Of the enrolled subjects, approximately 81% were males and 6% had symptomatic PAD with IC, 4% had diabetes, and 8% had coronary artery disease with previous bypass surgery or angioplasty at the time of enrollment. The mean baseline LDL-C level was 188.3 ± 25.9 mg/dL. Subjects were followed prospectively for a median of 5.4 years. The 4S study reported a 38% risk reduction in new or worsening IC in the treatment arm [relative risk (RR) 0.62 (0.44–0.88), *p* = 0.008]. There was a 28% reduction in cerebrovascular event rate indicating that statins likely have a generalized plaque-stabilizing effect that is not confined to the coronary arterial bed.

The next study to show a similar benefit of statins was the Heart Protection Study (HPS) Collaborative [28], which enrolled 6748 patients with PAD and randomly assigned them to receive either simvastatin 40 mg or placebo. The mean follow-up period for this study was 5 years. Investigators found an overall 16% relative reduction in the rate of a first peripheral vascular event following randomization (*p* = 0.006). Furthermore, there was a 22% relative risk reduction in the rate of first vascular events (*p* ≤ 0.0001) and a 20% relative risk reduction in non-coronary revascularization in PAD patients (*p* = 0.002). These benefits were independent of the pre-treatment lipid levels and the extent of PAD. The absolute reduction in major vascular events at baseline was found to be greater in patients with PAD (63 [SE 11] per 1000) than in those without (50 [SE 7] per 1000), reflecting a greater absolute reduction in revascularization among participants with PAD (42 [SE 9] per 1000) compared to those without (19 [SE 5] per 1000).

In a large analysis of benefits of statins in PAD, Kumbhani et al., analyzed 5861 patients with PAD enrolled in the REACH study (REduction of Atherothrombosis for Continued Health) [29] and reported on the rates of major adverse limb events (MALE) (defined as worsening claudication or a new episode of CLTI, any percutaneous or surgical revascularization, or amputation). They found an 18% relative risk reduction in MALE [hazard ratio (HR) 0.82, *p* = 0.0013] and 17% lower risk in the combined endpoint of CV death, non-fatal MI, or non-fatal stroke [hazard ratio (HR) 0.83, *p* = 0.01].

The first line treatment in patients with critical limb ischemia (CRITISCH) was an observational registry [30] that analyzed PAD outcomes and benefits of statin treatment. Their prospective observational cohort included 445 patients with CLTI who were on statin therapy and 371 patients who were not on statin therapy. Over a median follow-up period of 2 years, authors observed lower rates of amputation-free survival (HR 0.45, *p* = 0.001) and lower odds of major adverse CV and cerebral events [odds ratio (OR) 0.41, *p* = 0.001] in the group who were on statin therapy.

Similarly, the EUCLID trial (Examining the Use of Ticagrelor in Peripheral Artery Disease) [31] noted that the patients with a major amputation had a lower prevalence of statin use as compared to those who did not have an amputation (53% vs. 73.6%, *p* ≤ 0.001). They reported a lower rate of major amputation associated with statin use in both the overall PAD patient cohort (HR = 0.52, *p* < 0.001) and patients stratified by baseline CLTI status (HR = 0.47 *p* < 0.001).

In another large analysis of statin benefit, Arya et al., studied 155,647 veterans with PAD over a median follow-up period of 5.9 years and evaluated lower extremity (LE) amputations and mortality. Statin intensity exposure (high-intensity statin versus low-to-moderate–intensity statin versus antiplatelet therapy but no statin use) was determined within 1 year of diagnosis of PAD. Authors reported a significant reduction in mortality and amputation risk, respectively, in the low-to-moderate–intensity statin group (HR, 0.83; 95% CI, 0.81–0.85 and HR, 0.76; 95% CI, 0.72–0.80) when compared to antiplatelets only. High-intensity statins had a more significant benefit for both outcomes with a 30% risk reduction in mortality (HR, 0.70; 95% CI, 0.67–0.73) and a 39% risk reduction in amputation risk (HR, 0.61; 95% CI, 0.56–0.66) when compared to the antiplatelet-only group. In a 3-level propensity score-matched analysis comparison, 30,780 patients were matched in a 1:1:1 high-intensity statin, low-to-moderate–intensity statin, and active comparator group (antiplatelet drugs only). There was a statistically significant reduction in amputation risk (crude HR, 0.69; 0.61–0.76 and adjusted HR, 0.60; 0.52–0.69) and all-cause mortality (crude HR, 0.72; 0.68–0.76 and adjusted HR, 0.70; 0.66–0.75) for high-intensity statin users when compared to those taking only antiplatelet medications. There was a modest but statistically significant reduction in amputations (crude HR, 0.84; 0.75–0.93 and adjusted HR, 0.80; 0.70–0.91) and mortality (crude HR, 0.83; 0.79–0.88 and adjusted HR, 0.80; 0.75–0.85) for low-to-moderate–intensity statin users when compared to those taking only antiplatelet medications [32]. The risk reduction in amputation and mortality outcomes with high-intensity statins continued to be significant after propensity score matching and sensitivity and subgroup analyses.

Another observational study of 69,332 patients in Taiwan with diabetes and PAD assessed risk reduction in lower extremity amputations with statin use. Authors found a significantly lower risk of lower extremity amputation (HR 0.75, CI 0.62–0.90) and total amputations (HR 0.58, CI 0.36–0.93) among patients on statins as compared to those not on statins [33].

A Cochrane meta-analysis of 18 trials consisting of 10,049 patients with PAD was reported in 2009—the overall results surprisingly revealed no significant association of lipid-lowering treatment with either mortality or CV outcomes. However, after excluding an outlier study, the modified results showed a significant reduction in CV events (OR 0.74, CI 0.55–0.98) [34]. The study that was excluded in the modified analysis was the PQRST trial, which showed not only a reduction in total cholesterol and LDL-C but also high-density lipoprotein cholesterol (HDL-C) with probucol, the lipid-lowering agent studied in that trial [35]. Probucol may have had detrimental effects on outcomes via other mechanisms.

In a recent meta-analysis by Pastori et al., regarding statins and MALE in patients with PAD, the authors analyzed 51 studies that included 138,060 patients with PAD. Patients on statins had a 30% reduction in MALE (HR 0.70, CI 0.61–0.81) and a 35% reduction in amputations (HR 0.65, CI 0.52–0.82). The statin group also had a lower risk of all-cause mortality (HR 0.61, CI 0.54–0.68), CV death (HR 0.59, CI 0.46–0.78), composite CV endpoints (HR 0.66, CI 0.59–0.74), and ischemic stroke (HR 0.72, CI 0.62–0.83) [36]. Kokkinidis et al., conducted a systematic review and meta-analysis of 19 studies, including 26,985 patients with CLTI, and evaluated the effect of statin therapy on CV and limb events. Patients on statin therapy had a reduction in risk of major adverse CV and cerebral events (HR 0.5, CI 0.39–0.66, I^2^ = 0) and fatal events (HR 0.62, CI 0.52–0.75, I^2^ = 41.2) with a 25% decreased risk of amputation (HR 0.75, CI 0.59–0.95) [37].

Statins have also been shown to improve walking distance. Mondillo et al., reported a 90-m increase in pain-free walking distance in PAD patients after 6 months of simvastatin therapy (*p* < 0.005) [38]. In addition to increasing pain-free walking distance [39], statins have been shown to increase total walking distance [40,41,42,43,44], reduce severity of IC [45] and improve overall quality of life [43,46,47], especially when combined with an exercise regimen [48].

Multiple studies have described the benefits of statins in patients with established PAD or at moderate to high risk of vascular events as described above. However, until the last decade, the role of statin therapy in patients at lower risk of vascular events was not known. The Cholesterol Treatment Trialists published a meta-analysis of 27 randomized trials in 2012. Based on their baseline risk of 5-year major vascular events ranging from <5% to ≥30% on control therapy (no statin or low-intensity statin), participants were sorted into five categories. Major vascular events included major coronary events (nonfatal MI or coronary death), strokes, or coronary revascularization. Authors found a proportional reduction in major vascular events that was at least as high in the two lowest risk categories as in the higher risk categories (RR per 1 mmol/L reduction from lowest to highest risk: 0.62 [99% CI 0.47–0.81] for the <5% risk group, 0.69 [99% CI 0.60–0.79] for the ≥5% to <10% risk group, 0.79 [99% CI 0.74–0.85] for the ≥10% to <20% risk group, 0.81 [99% CI 0.77–0.86] for the ≥20% to <30% risk group, and 0.79 [99% CI 0.74–0.84] for the ≥30% risk group; trend *p* = 0.04). Furthermore, the study found that there were significant reductions in major coronary events (RR 0.57, 99% CI 0.36–0.89, *p* = 0.0012 and 0.61, 99% CI 0.50–0.74, *p* < 0.0001) and in coronary revascularizations (RR 0.52, 99% CI 0.35–0.75 and 0.63, 99% CI 0.51–0.79; both *p* < 0.0001) in the two lowest risk category groups, respectively. Among participants with 5-year risk of major vascular events <10%, risk reduction in stroke (RR per 1.0 mmol/L LDL-C reduction 0.76, 99% CI 0.61–0.95, *p* = 0.0012) was similar to that seen in higher risk categories (trend *p* = 0.30). Even among participants without a history of vascular disease, statins reduced the risks of vascular (RR per 1.0 mmol/L LDL-C reduction 0.85, 95% CI 0.77–0.95) and all-cause mortality (RR 0.91, 95% CI 0.85–0.97). Authors found no evidence that reduction in LDL-C with a statin increased cancer incidence (RR per 1.0 mmol/L LDL-C reduction 1.00, 95% CI 0.96–1.04), cancer mortality (RR 0.99, 95% CI 0.93–1.06), or other non-vascular mortality. The study concluded that, among individuals with a 5-year risk of major vascular events lower than 10%, each 1 mmol/L (~38 mg/dL) reduction in LDL-C produced an absolute reduction in major vascular events of about 11 per 1000 over 5 years. Further, the proportional reduction in events in patients with established vascular disease (which included patients with PAD) was similar to those without established vascular disease; however, absolute event rates are higher in those with established vascular disease, suggesting a larger absolute benefit with statin therapy in this patient population. This large analysis thus firmly established that the benefit of statins exceeded the risk of therapy [49].

### 4.3. Current Guidelines

**Figure 3:**
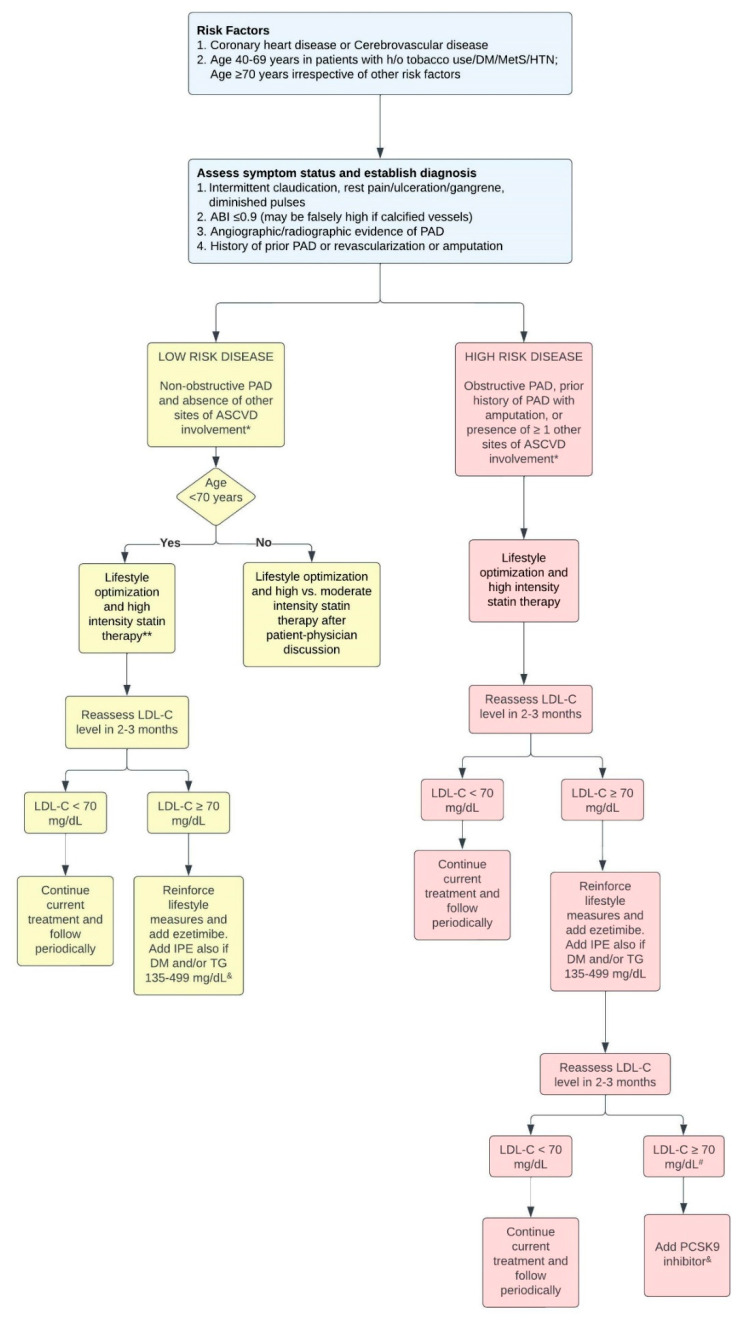
University of Louisville approach to PAD treatment based on current guidelines. * ASCVD disease includes CAD, PAD, TIA, stroke, history of myocardial infarction, aortic aneurysm, carotid atherosclerotic disease, history of coronary artery bypass grafting, or percutaneous coronary intervention. Risk factors include HTN, HLD, DM, age ≥70 years, LDL-C >100 mg/dL, high-sensitivity CRP > 2 mg/dL, chronic kidney disease, and Lp(a) > 125 nmol/L. ** High-intensity statins include atorvastatin 40–80 mg and rosuvastatin 20–40 mg. Moderate-intensity statins include atorvastatin 10–20 mg, rosuvastatin 5–10 mg, simvastatin 20–40 mg, and pravastatin 40–80 mg. ^&^ Bempedoic acid may also be considered, but it is pending data from an ongoing cardiovascular outcomes trial. ^#^ LDL-C > 55 mg/dL if in a very-high risk group as defined in the 2019 ESC guidelines [7]. CAD: Coronary artery disease, PAD: Peripheral artery disease, HTN: Hypertension, HLD: Hyperlipidemia, DM: Diabetes Mellitus, MetS: Metabolic syndrome, TIA: Transient ischemic attack, LDL-C: Low-density lipoprotein cholesterol, ASCVD: Atherosclerotic cardiovascular disease, TG: Triglycerides, IPE: icosapent ethyl, PCSK9: Proprotein Convertase Subtilisin/Kexin Type 9.

Current European guidelines recommend achieving a ≥50% reduction in blood LDL-C levels to <55 mg/dL in patients with atherosclerotic cardiovascular disease (ASCVD), which includes those with PAD. A high-intensity statin, at the highest tolerated dose, should be prescribed as first-line therapy given the evidence showing reductions in MALE and MACE. Addition of ezetimibe or proprotein convertase subtilisin/kexin type 9 (PCSK9) inhibitors is recommended if the LDL-C remains above goal (based on the degree of risk, ESC guidelines give a goal of either <55 or <40 mg/dL in those with recurrent ASCVD events) [7]. The multi-society guidelines for management of patients in 2018 classified PAD as an ASCVD equivalent alongside MI, stroke, and CAD [50]. The 2016 AHA/ACC guidelines on PAD management also give statins a Class IA recommendation in patients with PAD; although PAD itself will not make a patient eligible for LDL-C lowering to <70 mg/dL per the ACC/AHA guidelines, it is one of the major ASCVD events that define the “very high-risk ASCVD” category [6]. Based on these guidelines, we propose a flowchart to approach lipid management in patients with PAD (Figure 3).

### 4.4. Underuse of Statins in PAD

Unfortunately, despite the substantial database for benefit of statins and other lipid-lowering therapies in PAD, there remains suboptimal usage of these drugs in PAD patients [51] The PREVENT III trial noted that 54% of patients with CLTI were not on any lipid-lowering therapy [52]. The observational study by Arya et al., discussed above also reported similar underuse of statins—the use of high-intensity statins was the lowest (6%) in PAD patients, as compared to patients with either coronary or carotid artery disease (18.4%). This highlights clinician and patient underappreciation of PAD as a systemic atherosclerotic disease. In the observational study of Hess et al., that included 250,103 patients with PAD, ~40% were not on any lipid-lowering therapy despite the observed increased risk of MALE and MACE. Similar to the prior observation, lipid-lowering therapies were used less often and at lower doses for PAD patients in contrast to coronary artery disease (CAD) patients who were more frequently prescribed high-intensity statins [53]. Colantonio et al., also noted similar findings in their database—although the risk of CV events in patients with PAD was comparable to those with CAD (HR 0.91, CI 0.86–0.95), the former was significantly less likely to have been prescribed statins. In addition, having coexisting CAD or cerebrovascular disease increased the rate of statin prescription in the PAD population (statins were prescribed to 33.9% of patients with PAD only; CAD only: 51.7%, PAD + cerebrovascular disease: 46.5%; PAD + CAD: 50.2%; PAD + CAD + cerebrovascular disease: 56.2%) [54].

## 5. Role of PCSK9 Inhibitors in Patients with PAD

### 5.1. Mechanism of Action of PCSK9 Inhibitors

PSCK9 is a serine protease involved in the degradation of LDL receptors on hepatocytes, which leads to higher plasma LDL-C levels. PSCK9 inhibitors, such as alirocumab and evolocumab, are humanized monoclonal antibodies that bind the PSCK9 protein, inhibiting its attachment to the LDL receptor and, therefore, decrease plasma LDL-C concentrations [55]. Statins upregulate PCSK9 levels—thus, combined statin and PCSK9 inhibitor therapy is especially beneficial in patients who are unable to reach therapeutic LDL-C lowering with statin therapy alone [56].

### 5.2. Clinical Outcomes of PCSK9 Inhibitors in Patients with PAD

The FOURIER (Further Cardiovascular Outcomes Research with PCSK9 Inhibition in Subjects with Elevated Risk) trial was a double-blinded randomized control trial that included 27,564 patients with stable atherosclerotic CV disease on statin therapy. Subjects were randomized to receive evolocumab (140 mg every 2 weeks or 420 mg monthly) vs. placebo. The FOURIER trial was continued till at least 1630 patients reached the composite endpoint of CV death, MI, or stroke, thus providing a 90% power to detect a relative reduction of ≥15% for the secondary endpoint [57]. Compared to placebo, patients receiving evolocumab were noted to have a 19% reduction in first acute arterial events (HR 0.84, CI 0.74–0.88) and a 24% reduction in total event rate (HR 0.76, CI 0.69–0.85) in the first year. Individual event rates of acute coronary events, peripheral vascular events, and cerebrovascular events also showed a decline, with a HR of 0.83 (CI 0.75–0.91), 0.58 (CI 0.38–0.88) and 0.77 (CI 0.65–0.92), respectively [58].

Bonaca et. al., conducted a subgroup analysis of 3647 patients with PAD in this trial with the primary endpoint being a composite of CV death, MI, stroke, hospital admission for unstable angina, or coronary revascularization. They evaluated MALE, ALI, major amputation, or urgent peripheral revascularization for ischemia. The evolocumab group had a 21% reduction in the primary endpoint (HR 0.79, CI 0.66–0.94, *p* = 0.0098) and a 42% reduction (HR 0.58, CI 0.38–0.88, *p* = 0.0093) in MALE [59]. Additionally, the absolute risk reduction in the primary endpoint was higher in the PAD subgroup than in those without PAD (3.5% vs. 1.6%) [59,60].

The ODYSSEY outcomes (evaluation of cardiovascular outcomes after an acute coronary syndrome during treatment with alirocumab) trial was a multicenter double-blinded placebo-controlled trial with a median duration of 2.8 years follow-up involving 18,924 patients with a history of an acute coronary syndrome in the last 12 months, LDL-C at least 70 mg/dL, or non-HDL-C at least 100 mg/dL/or apoB level of at least 80 mg/dL on high-intensity or maximally tolerated dose of statin. The primary endpoint of the study was a composite of death from coronary artery disease, nonfatal MI, fatal or nonfatal ischemic stroke, or unstable angina requiring hospitalization. The primary endpoint rate was reduced in the alirocumab group by 15% (HR 0.85, CI 0.78–0.93, *p* < 0.001) [61]. A subgroup analysis of the ODYSSEY outcomes trial investigated PAD events after ACS. Alirocumab was found to reduce the risk of PAD events (HR 0.69, 95% CI 0.54–0.89, *p* = 0.004) [61,62].

Based on the above data, current guidelines recommend consideration of PCSK9 monoclonal antibodies in patients who do not meet LDL-C treatment goals with dietary modification and other lipid-lowering therapies, such as maximally tolerated statin plus ezetimibe [50].

## 6. Role of Icosapent Ethyl (IPE) in PAD

IPE is an ethyl ester of eicosapentaenoic acid and reduces hepatic very low-density lipoprotein triglycerides (VLDL-TG) synthesis and/or secretion, thereby enhancing TG clearance from circulating VLDL particles. Patients with elevated TG levels are at increased risk for ischemic events due to an increase in remnant lipoproteins. The REDUCE-IT trial enrolled 8179 patients who were randomized to receive IPE vs. placebo. The primary endpoint of this study was a composite of CV death, nonfatal MI, nonfatal stroke, coronary revascularization, or unstable angina. Among patients with TG levels 135–400 mg/dL despite the use of statins, a primary end-point event occurred in 17.2% of the patients in the IPE group, as compared with 22.0% of the patients in the placebo group (HR, 0.75; 95%, CI 0.68 to 0.83; *p* < 0.001) [63]. Furthermore, the EVAPORATE substudy of coronary CT imaging of plaque features also demonstrated a significant regression of low-attenuation coronary plaque volume on multidetector CT in patients who were randomized to IPE compared to placebo, over 18 months [64].

Current guidelines recommend that if the TG levels remain high after diet and exercise, IPE may be added to statin for reduction in CV risk [65]; however, their specific benefit in PAD patients remains to be explored.

## 7. Role of Fibrates, Ezetimibe and Niacin in PAD

The role of fibrates, ezetimibe, and niacin in PAD has mostly been studied with these drugs being used as an adjunct to statin therapy. Sohn et al., compared outcomes for patients with diabetes taking statins to those taking non-statin lipid-lowering agents, such as fibrates, bile acid sequestrants, nicotinic acid, and cholesterol absorption inhibitors (including ezetimibe) and found that those patients taking non-statin lipid therapy did not show a reduction in LE amputation rates [66]. A study by West et al., showed that statin initiation with or without ezetimibe in statin-naïve patients halted the progression of PAD. When ezetimibe was added to patients previously on statins, PAD paradoxically progressed—the authors concluded that ezetimibe’s effect on PAD seemed to depend upon the relative timing of ezetimibe initiation with respect to statin therapy. However, this study had a few shortcomings, such as it was underpowered and small, 23% (20/87) of the patients initially enrolled in the study were not included in the final analysis, there was no placebo arm, and the endpoint was superficial femoral artery vessel wall plaque volume change over 2 years (as measured by MRI) rather than CV events or MALE [67].

## 8. Novel Drugs: Inclisiran

Inclisiran is a small interfering RNA that inhibits hepatic PCSK9 and upregulates the number of LDL-receptors on the hepatocytes. Inclisiran was approved in the European Union in 2020 for use in adults with primary hypercholesterolemia or mixed dyslipidemia based on the results of the ORION trials [68]. Inclisiran was shown to be effective in reducing LDL-C in patients with elevated LDL-C who were on maximally tolerated statin therapy. In a study conducted by Ray et al., 501 patients with high LDL-C levels (LDL-C > 70 mg/dL for patients with ASCVD or >100 mg/dL for those without) were randomly assigned to receive inclisiran or placebo. The primary endpoint was the change from baseline in LDL-C levels at 180 days. Safety data were available through day 210 and data on LDL-C and PCSK9 levels were available through day 240. At 180 days, the least-squares mean reductions in LDL-C levels ranged from 27.9 to 41.9% after a single dose of inclisiran and 35.5 to 52.6% after two doses (*p* < 0.001 for all comparisons with placebo). Greatest reduction in LDL-C levels was seen with the two-dose 300-mg inclisiran regimen; 48% of the patients who received this regimen had an LDL-C level below 50 mg/dL at 180 days. The reduction in LDL-C and PCSK9 levels from baseline brought about by inclisiran persisted even at day 240 irrespective of the initial regimen [69]. The ongoing ORION-4/TIMI 65 trial is studying CV outcomes in patients with ASCVD or at high risk. The effects of inclisiran in PAD remain to be explored.

## 9. Role of Apheresis in PAD

The LIPAD study enrolled 213 patients with symptomatic PAD and matched them to controls for sex, age, and presence of diabetes. Lipoprotein (a) [Lp(a)] concentrations above the 75th percentile of the entire cohort were significantly associated with PAD with an odds ratio of 3.73 even after adjustment for potential confounding factors, highlighting the role of elevated Lp(a) in PAD [70]. The role of lipoproteins in PAD was confirmed in the MESA trial [16]. Among the 4618 participants enrolled in this study, the mean age was 62 ± 10 years. Mean Lp(a) was 30 ± 32 mg/dL and median (interquartile range) was 18 (8–40 mg/dL), and 11% of all participants had established PAD at the time of enrollment. After adjustment for traditional CV disease risk factors and interleukin-6, fibrinogen, D-dimer, and homocysteine levels, authors found an association between logarithmic increase in Lp(a) levels and higher odds for PAD (OR, 1.12; 95% CI, 1.01–1.25). The EPIC-Norfolk study showed that Lp(a) levels in the highest quartile were more predictive of incident PAD (adjusted HR 2.06) compared to CAD (adjusted HR 1.33) [71].

Apheresis for high Lp(a) has been used in selective patients with ASCVD especially in Germany [72,73]; however, its effects of reducing Lp(a) levels have been studied more for MACE rather than MALE [74,75,76,77]. Prospective cohort studies strongly suggest a causal relationship between elevated Lp(a) levels and PAD. However, data on the efficacy of lipoprotein apheresis in patients with PAD and elevated Lp(a) are scarce [78]. The HORIZON trial is an ongoing clinical trial to assess the impact of LP(a) lowering with Pelacarsen (TQJ230) on MACE in patients with CV disease [79].

A summary of landmark studies on the role of lipid-lowering therapy in PAD is described below (Table 1).

## 10. Conclusions

The impact of traditional risk factors, such as age, diabetes, elevated blood pressure, smoking, and high levels of LDL-C and Lp(a) on the risk of developing atherosclerosis and PAD has been well established [80]. Based on the above studies, there is a clear benefit of lipid-lowering therapy in patients with PAD. However, this clinical entity, unfortunately, continues to be underdiagnosed and undertreated when compared to coronary and cerebrovascular atherosclerosis, and there is a significant racial and socioeconomic disparity in the diagnosis and management of PAD.

Advances in basic science over the last three decades have established a fundamental role for inflammation in mediating all stages of atherosclerosis from initiation through progression and, ultimately, the thrombotic complications of this disease [81,82]. Decreased levels of LDL-C, better blood pressure control, and lower incidence of smoking have brought about an evolution in the risk factor profile for development of atherosclerosis. There is a focus on triglyceride-rich lipoproteins in addition to LDL as culprits in atherosclerosis. Non-traditional risk factors for atherosclerosis, such as disturbed sleep, the gut microbiome, physical inactivity, air pollution, and environmental stress, have also gained consideration. Both traditional and emerging risk factors for atherosclerosis are tied together by inflammatory pathways and leukocytes that have been incriminated in altering the behavior of arterial wall cells [83]. The role of these novel, non-traditional risk factors in the development and progression of PAD remains to be explored.

Atherosclerosis and PAD bear significant morbidity and mortality and form an important part of contemporary clinical practice. Preventive strategies, such as diet and lifestyle modification and lipid-lowering pharmacotherapy, form the cornerstone of prevention and treatment of PAD and its complications.

## Figures and Tables

**Table 1:** Summary of landmark studies on the role of lipid-lowering therapy in PAD.

Study (Year)	Study Design	Sample Size	Patient Population	Intervention	Median Follow Up Time	Outcome	Interpretation
Pedersen (4S Study), 1994 [27]	Randomized controlled trial (RCT)	4444	MI or angina pectoris, serum cholesterol between 213–309 mg/dL	Simvastatin 20–40 mg daily vs. placebo	5.4 years	Intermittent claudication	Statin therapy may help in plaque stabilization and may also have a general anti-atherosclerotic effect.
Heart Protection Study Collaborative Group, 2002 [28]	RCT	6748	History of PAD, CAD, stroke, diabetes, treated hypertension	Simvastatin 40 vs. placebo	5 years	First major vascular event	Statin treatment showed improvement in MACE, overall revascularizations in all patients with PAD irrespective of their pre-treatment lipid levels.
Kumbhani (REACH registry), 2014 [29]	Retrospective review	5861	Symptomatic PAD	Statin vs. no statin	4 years	Primary adverse limb events, primary endpoints of CV death, non-fatal MI, or non-fatal stroke	Patients taking statins had significantly lower risk of MACE and MALE at 4 years.
Stavroulakis (CRITISCH registry), 2017 [30]	Retrospective analysis of prospectively collected data	816	Presence of new onset CLTI	Statin vs. no statin	2 years	MACE and cerebral events, amputation free survival	Statin treatment showed improvement in amputation-free survival and overall cardio/cerebrovascular events in patients with new onset CLTI
Arya, 2018 [32]	Retrospective observational cohort study	155,647	Incident PAD	High-intensity statin therapy vs. low-to-moderate–intensity statin vs. other or no therapies for PAD	5.9 years	High-intensity statin vs. antiplatelet therapy: Amputation rates, low-to-moderate–intensity statins vs. antiplatelet therapy only: Amputation rates	Statin therapy showed reduced risk of amputation and overall morality as compared to antiplatelet therapy and high-,intensity statin therapy noted more pronounced improvement in comparison to low–moderate-intensity statins.
Hsu, 2017 [33]	Retrospective observational cohort study	69,332	≥20 years old with diabetes and PAD	Statin vs. non-statin lipid treatments vs. non-user group	5.7 years	Statin vs. non statin user, incident LE amputation risk, in-hospital CV death, and all-cause mortality	Statin therapy noted decreased risk of incident and total amputations in patients with diabetes and PAD. It also showed improvement in CV and mortality outcomes.
Aung, 2007 [34]	Cochrane meta-analysis	10,049	PAD	Lipid lowering treatment vs. none	NA	Total CV events, total coronary events	Lipid lowering therapy improves CV outcomes in patients with PAD.
Pastori, 2020 [36]	Meta-analysis	138,060	PAD	Statins vs. no statins	NA	MALE, amputations, all-cause mortality, CV deaths, and ischemic stroke	Statin therapy in PAD patients reduces adverse limb outcomes and cardio and cerebrovascular events, as well as overall mortality.
Kokkinidis, 2020 [37]	Meta-analysis	26,985	Existing CLTI	Statins vs. no statins	NA	Major adverse CV and cerebral events, amputations	Statin use can decrease overall CV and cerebral outcomes in addition to overall mortality. It also might decrease amputation rates, but the data was noted to have significant heterogeneity.
Mondillo, 2003 [38]	RCT	86	PAD (Fontaine stage II), intermittent claudication and cholesterol levels >220 mg/dL	Simvastatin 40 mg daily vs. placebo	0, 3 and 6 months	Pain-free walking distance at 6 months, total walking distance	Simvastatin therapy in patients with pre-existing PAD, IC and hypercholesterolemia showed improvement in 6-month pain-free walking distance and total walking distance.
Mihaylova (Cholesterol Treatment Trialists group), 2012 [49]	Meta-analysis	134,537	NA	Statin vs. no statin	NA	Major vascular event in patients at low 5-year risk of major vascular event	Even in patients with low 5-year major vascular event risk, low-dose statins showed absolute reduction in major vascular events.
Oyama (FOURIER), 2018 [58] Bonaca (FOURIER Insights), 2021 [59]	RCT	27,564	Prior MI, non-hemorrhagic stroke, or symptomatic PAD, with LDL ≥70 mg/dL or non-HDL-C ≥100 mg/dL while on high- or moderate-intensity statin +/− ezetimibe	Evolucumab vs. placebo	2.2 years	First acute arterial event, total event rate, ACS, peripheral vascular events, cerebrovascular events	Addition of evolocumab over statin therapy with or without ezetimibe improves vascular outcomes in all territories.
Schwartz (ODYSSEY OUTCOMES), 2018, 2020 [61,62]	RCT	18,924	History of ACS in last 12 months, LDL-C ≥70 mg/dL, HDL-C at least 100 mg/dL, apoB at least 80 mg/dL on high-intensity or maximally tolerated dose of statin	Alirocumab vs. placebo	2.8 years	Composite death from CAD, non-fatal MI, fatal or non-fatal ischemic stroke, or unstable angina requiring hospitalization	Alirocumab also shows improvement in overall CV outcomes when prescribed in addition to maximally tolerated or high-dose statin therapy. It also showed improvement in PAD and venous thromboembolism outcomes in these patients.

## Data Availability

Not applicable.
